# A Software Product Line Process to Develop Agents for the IoT

**DOI:** 10.3390/s150715640

**Published:** 2015-07-01

**Authors:** Inmaculada Ayala, Mercedes Amor, Lidia Fuentes, José M. Troya

**Affiliations:** Departamento de Lenguajes y Ciencias de la Computación, Andalucía Tech, Universidad de Málaga, Campus de Teatinos s/n, 29071 Málaga, Spain; E-Mails: pinilla@lcc.uma.es (M.A.); lff@lcc.uma.es (L.F.); troya@lcc.uma.es (J.M.T.)

**Keywords:** agents, SPL, CVL, IoT, variability modeling

## Abstract

One of the most important challenges of this decade is the Internet of Things (IoT), which aims to enable things to be connected anytime, anyplace, with anything and anyone, ideally using any path/network and any service. IoT systems are usually composed of heterogeneous and interconnected lightweight devices that support applications that are subject to change in their external environment and in the functioning of these devices. The management of the variability of these changes, autonomously, is a challenge in the development of these systems. Agents are a good option for developing self-managed IoT systems due to their distributed nature, context-awareness and self-adaptation. Our goal is to enhance the development of IoT applications using agents and software product lines (SPL). Specifically, we propose to use Self-StarMASMAS, multi-agent system) agents and to define an SPL process using the Common Variability Language. In this contribution, we propose an SPL process for Self-StarMAS, paying particular attention to agents embedded in sensor motes.

## 1. Introduction

One of the most important challenges of this decade is to accomplish the Internet of Things (IoT) or the integration of devices from the real world inside the Internet [1,2]. The IoT is a concept and a paradigm that considers pervasive presence in the environment of a variety of things (also called objects) that are able to interact with each other and cooperate to create new applications and services and reach common goals. IoT systems aim to create smart environments that make energy, transport, cities and many other areas more intelligent.

The goal of the IoT is to enable things to be connected anytime, anyplace, with anything and anyone ideally using any connectivity technology and any service. Applications of IoT are numerous and varied, propagating into almost all domains of everyday life and activities of individuals and organizations. The challenges are numerous, varied and often related to a particular domain or the context in which an application is used. However, the IoT vision increases the scale, complexity and heterogeneity of the existing computing and communication systems involved in the IoT. Showing an autonomous behavior is thus a relevant property for IoT systems to have. Moreover, this should be considered from the very early phases of IoT systems' implementation, from early requirements to the deployment of devices, infrastructures and services. One property that characterizes IoT systems is that they are composed of globally-connected, highly dynamic and interactive networks of physical and virtual devices [1], which have to react to variable and continuous changes in their context. This means that IoT systems need to work as self-managed systems to effectively manage context changes. Self-management must be able to cope with the variability of IoT systems, which are subject to dynamic changes in their environment, inside the devices that comprise the system (e.g., resource scarcity) or in the global network that comprises the IoT application (e.g., failure of one of the nodes). Therefore, one of the common challenges in the development of IoT applications is to effectively manage these changes autonomously, despite their variability and the complexity.

The distributed and variable nature of IoT systems can be managed with the autonomy, the context-awareness and the self-adaptation properties of software agents, supporting the development of autonomous IoT applications. The most suitable solution is to use agents as the building blocks of an IoT application, providing the functionality of the distributed application and performing self-management tasks. The autonomy property inherent to software agents makes them a suitable choice for developing self-managed IoT systems. By embedding agents in the devices that compose the IoT, it is possible to realize a decentralized system with self-management capacities. There are others that propose the use of agents for the IoT [3–5], but they do not consider self-management. The proactive and autonomous behavior of agents, usually modeled in terms of goals, means that they are able to be aware of and adapt to the particular context in which they are embedded according to a set of self-management goals. These self-management goals endow agents in IoT systems with the ability to adapt their behavior to run continuously in adverse conditions, such as changing environments, partial subsystem failures and changing user needs. Often, they must run unattended without interruption. Agents running in smart devices also need to adapt their overall system behavior to energy levels and varying quality in network connection. This allows an effective reconfiguration management of IoT systems.

Therefore, in this scenario, new challenges arise for software developers. There is a need to produce software agents for the IoT that are capable of evolving and adapting to different system management requirements while meeting the application goal for which they were intended. The developments of such IoT applications have several features in common, but also have a high variability due to the heterogeneity of devices and technologies and networking issues involved in the IoT scenario. However, current and traditional agent development processes lack the necessary mechanisms to tackle specific management of components between different applications of the IoT, bearing in mind the inherent variability of these systems.

Our goal is to enhance the development process of self-managed IoT systems based on software agents with the help of product line engineering, a well-known and widely-used technology in the industry [6,7]. Product line engineering is fundamentally a reuse-oriented approach that uses a common reference architecture to develop a well-designed set of assets that fit together, the software product line (SPL). The use of SPLs has achieved improvements in time, cost reduction and quality of software [7].

A fundamental principle of SPLs is variability management [6], which involves separating the product line into three parts (common components, parts common to some, but not all products, and individual products with their own specific requirements), which are managed separately during system development. The benefits of SPL are provided by the reusability of the characteristics (features) common and variable, embodied in architectural elements during the development of a new product or configuration. Today, this technology is being successfully applied in many application domains, including the development of multi-agent systems. The integration of these two technologies together is known as MAS-PL (multi-agent system product lines) [8–13]. However, previous MAS-PL approaches have not focused on solutions for the IoT, and they have been restricted to the analysis phases of SPL engineering. In addition, only [9] considers self-management in the context of the SPL. The SPL employs a two-life-cycle approach that separates domain and application engineering. While the first part is concerned with analyzing the problem solution as a product line in order to produce any common (and reusable) variable parts, in the second part, the application engineering involves creating product-specific parts and integrating all aspects of individual products. Specifically, we intend to define an SPL process using the Common Variability Language (CVL) [14] for Self-StarMAS [15] agents.

Self-StarMAS is a family of aspect-oriented agents with self-management capabilities, which is based on the Malaca agent architecture [16]. We have chosen this agent technology because its agents can be embedded in typical devices of the IoT, like sensors, and can communicate through different agent platforms and network technologies. Moreover, its self-management capability can manage variability at runtime [17]. Using Self-StarMAS agents, we can develop the IoT application as a community of cooperating self-managed agents. For its part, CVL is a domain-independent language for specifying and resolving variability. This language is a proposed standard of the Object Management Group (OMG), and it has been applied to application domains, similar to the IoT [18,19]. CVL provides our development process with appropriate reuse mechanisms to develop a family of Self-StarMAS agents in the IoT, paying particular attention to the inherent variability of these systems.

This paper is organized as follows: Section 2 presents Self-StarMAS agents. Section 3 overviews CVL. Section 4 presents our main contribution, the SPL process for Self-StarMAS agents. Section 5 presents the domain engineering of our SPL process, while Section 6 presents the application engineering for a case study in smart shopping. Section 7 explains the background in related work. Section 8 summarizes lessons learned; and the paper closes with the conclusion and a discussion of future work in Section 9.

## 2. Self-StarMAS Agents

Self-StarMAS is a FIPA-compliant [20] agent system, which adapts and extends standard agent technologies to help in the development of IoT applications. In this system, we can distinguish two parts: Self-StarMAS [17], a set of cooperating agents developed for lightweight devices; and the agent platform where the agents are deployed, which is the middleware that provides a set of (FIPA-compliant) services for those Self-StarMAS agents running in several lightweight devices (*i.e.*, the agent platform).

The different versions of Self-StarMAS agents are embedded in Android devices, Sun Small Programmable Object Technology (Sun SPOTs) [21] and Libelium waspmotes [22]. A distinguishing feature of these agents is that they have self-management capabilities, adapted to the resources of the devices where agents are embedded [17]. Self-StarMAS agents for Android support goal-oriented and reactive reasoning engines. Self-StarMAS agents can be executed on top of different agent platforms and can use different transport protocols, by simply using the correct distribution plug-in. For instance, by using the Jade-Leap plug-in, these agents can communicate with other agents registered in a Jade-Leap agent platform. However, current agent platforms for lightweight devices are not entirely capable of managing both device and transport protocol heterogeneity, which means that they are unable to ensure communication interoperability in IoT systems. With this premise, the Sol agent platform [23] has been created to cope with these limitations. The work of Self-StarMAS with Jade-Leap and Sol has been validated in previous work [17,23,24], showing good results with regard to memory occupation, message latency and resource consumption.

FIPA-based agents require a set of services from the FIPA agent platform that are related to the transportation of messages between agents and with the discovering of agents and services. Sol is a FIPA-compliant agent platform particularly suitable for developing applications for IoT environments. This agent platform acts as an agent-based middleware that provides a set of services for the agents and behaves as a gateway to support communication heterogeneity.

The main features of this agent platform are the support for communication of agents in heterogeneous devices, coping with heterogeneous transport protocols (WiFi, Bluetooth and ZigBee) and group communications, which are often required by pervasive systems. Additionally, Sol has remote nodes (Sol clients), which communicate with the node in which Sol is running. The development of these clients has been necessary for the implementation of applications distributed over wide areas. Sol clients support devices with low-range communication technology, such as mobile phones that use Bluetooth, Sun SPOTs and Libelium waspmotes. These clients can run in desktop computers and in Meshlium multi-protocol routers [25].

In summary, the combined use of Self-StarMAS and Sol provides the necessary means for developing IoT applications. Self-StarMAS agents can take advantage of the Sol agent platform, to communicate through different transport protocols and send multicast messages to a group of related agents. With this approach, the functionality of the IoT applications is decomposed into a set of Self-StarMAS cooperating agents that use the Sol agent platform for location and communication between agents.

## 3. Software Product Lines and Common Variability Language: Overview

SPLs refer to the methods, tools and techniques for creating and maintaining a collection of similar software systems from a shared set of software assets [26]. A key aspect of SPL is the variability modeling or the description of more than one variant of a system. The variability of SPLs can be specified using different modeling languages. Although traditionally, feature models (FMs) have been very popular in the SPL community over the last decade, recently, the Common Variability Language (CVL) [14] has been proposed as a standard. Both variability languages can be used in our proposal, but in this approach, we have opted to use CVL.

**Figure 1:**
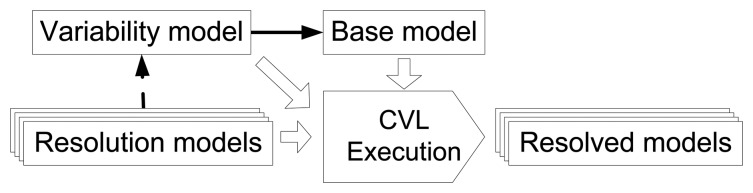
Common Variability Language (CVL) as specified in [14].

CVL is a domain-independent language for specifying and resolving variability over any instance of any language defined using a meta object facility (MOF)-based metamodel (e.g., the Unified Modeling Language (UML) [27] or a domain-specific language (DSL) [28] defined in Ecore [29]. The CVL overview and related terms are denoted in Figure 1. The instance of the MOF-based metamodel is referred to as the base model. CVL allows us to specify the variabilities of the base model in a CVL variability model.

The variability model is a specification in CVL of the base model variabilities and is divided into two parts. The first part is defined over the base model and marks its variation points. There are different types of variation points: to indicate the existence of an element; the substitution of a single object or an entire model fragment for another; and the value assignment of a particular slot of the model. In addition, variation points can be grouped into configurable units.

The second part of the variability model defines the relationships between the variation points of the base model, by means of variability specifications (VSpec), which are organized using a hierarchical structure called a VSpec tree. The sub-tree under a VSpec means that the resolution of this VSpec imposes certain constraints on the resolutions of the VSpec in its sub-tree. These constraints will be explained in the following sections with respect to the VSpec tree of our multi-agent system. Additionally, it is possible to specify explicit constraints, also known as cross-tree constraints. VSpecs are abstract and do not define which base model elements are involved nor how they are affected. The effect of the variability model on the base model is specified by binding variation points, which relates the base model and the VSpec tree.

Once the variability model and the base model have been defined, a set of VSpecs are selected from the VSpec tree. This selection of VSpecs is referred to as the resolution model. Then, the CVL tool is executed, and it obtains the resolved model, a product model, fully described in the MOF-based metamodel, which is a variation of the base model according to the choices that have been made in the resolution model.

## 4. CVL Development Process for Self-StarMAS Agents

**Figure 2:**
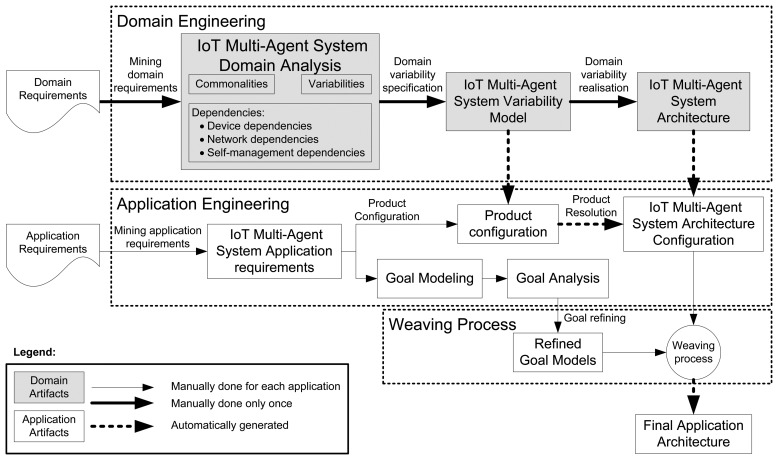
SPL process for Self-StarMAS agents. MAS, multi-agent system.

As stated in the Introduction, our goal is to develop an appropriate reuse mechanism to develop a family of systems in the IoT, paying particular attention to the inherent variability of IoT systems and in their self-management. Specifically, we propose an SPL process for Self-StarMAS agents instantiated for CVL (the process is shown in Figure 2). A typical SPL process comprises two phases, namely the domain engineering and the application engineering. Our domain engineering phase, which is done once, defines and realizes the commonality and the variability of multi-agent systems in the IoT as an SPL. On the other hand, the application engineering phase of the SPL deals with the multi-agent system-based applications for the IoT, which are designed and built by reusing domain artifacts and exploiting product line engineering [30] from the previous process. Our SPL process (see Figure 2) introduces an extra process, called the weaving process, that weaves the goal model of the agents with the architecture generated in the application engineering phase. Therefore, in order to apply our proposal, agent developers only have to focus on the application engineering process. In this phase, they use application requirements to accomplish two tasks, the selection of a product configuration of the SPL and the modeling and analysis of the agent goals. The final application architecture is automatically derived from these models.

In the following sections, the different activities of our SPL process for a case study in the IoT domain are detailed. The use of CVL in our development process enables the management of the variability presented in this domain. Additionally, SPLs provide specific mechanisms to manage the dependencies between the different concerns from the requirement stage. Therefore, the CVL process presented in this paper has been customized for the development of Self-StarMAS agents. In order to incorporate Self-StarMAS into an SPL approach, its architecture has been refactored. The different versions of Self-StarMAS agents for heterogeneous devices and network technologies with their different levels of cognition and self-management are now contained in the SPL architecture.

## 5. Domain Engineering

This section describes the first phase of our SPL process, the domain engineering phase (see the top of Figure 2), which identifies and models the agents for the IoT, their commonalities and variabilities, as well as the dependencies between them. As stated, this phase is accomplished using CVL, and our MOF-based language is the Unified Modeling Language [27]. This phase is divided into three different processes: IoT multi-agent system domain analysis, generation of the IoT multi-agent system variability model and definition of IoT multi-agent system architecture.

### 5.1. IoT Multi-Agent System Domain Analysis

The IoT multi-agent system domain analysis process encompasses all of the activities for eliciting and documenting the common and variable requirements of agents in the IoT. To do so, we have revised and analyzed different surveys in the domain of the IoT [1,31–33] and existing agent technologies suitable for the IoT [24,34]. From this analysis, we can derive the heterogeneity of target devices (IoT applications are comprised by different devices that can be anything from personal computers to sensors). Additionally, these applications use different network technologies, such as WiFi or NFC, and require self-management capacities for their correct functioning. Therefore, we can find many variation points irrespective of the device or the connectivity required by a IoT system. Our study on agent technology comes from a domain related to the IoT, the ambient intelligence (AmI) domain. Agents for this domain are suitable for the development of IoT applications, and they vary from traditional agents with respect to their cognitive capacities and the device where the agent is embedded.

Our domain analysis classifies the set of features shown by IoT applications based on the agents involved (e.g., the device type in which they can be embedded, the device operating system or its connectivity facilities) and to subsequently identify their common and variable features. We must also take into account the different dependencies and constraints between the features of these applications (for instance, the sensor mote device implies the use of a reactive reasoning engine in the agent). This information is formally specified in the VSpec tree (described in the next section).

Finally, as part of this process, the architecture of already developed Self-StarMAS agents has also been reviewed. We have used Self-StarMAS to effectively implement AmI systems in different areas and with different application requirements. Therefore, our previous versions of the Self-StarMAS agents [35] have been adapted to new devices (*i.e.*, Symbian, Android, Sun SPOT, Libelium) and network technologies (*i.e.*, WiFi, Bluetooth, ZigBee, XBee, NFC) and extended with additional features, such as goal orientation [36] and self-management [17]. The inclusion of self-management has been especially challenging, because it entails the development of different variations or levels of self-management to meet different computational resources and cognitive capacities of agents and devices.

### 5.2. IoT Multi-Agent System Variability Model

**Figure 3:**
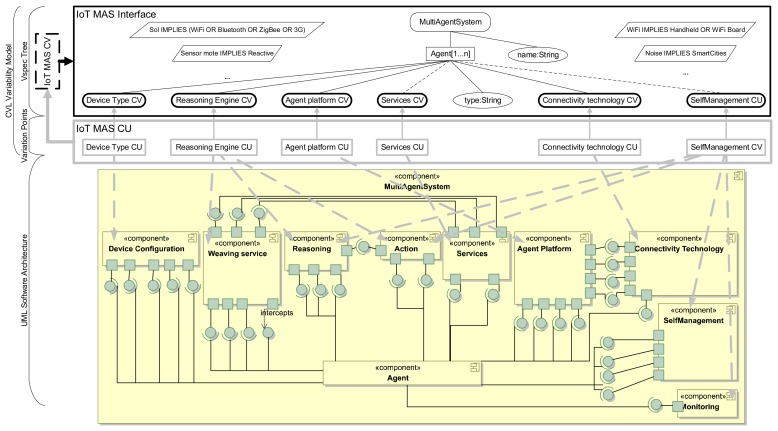
Complete variability model of the multi-agent system for the IoT in CVL.

Once the domain information has been captured in the analysis of the multi-agent system for the IoT, we define its variability model, which is specified using CVL. The VSpec tree of our variability model (see the top of Figure 3) is composed of three types of VSpec: choice, which represents a yes/no decision or elements that can be included or not in the resolution model (e.g., *MultiAgentSystemVSpec*); the variability classifier (*VClassifier*), whose resolution requires creating instances and then providing per-instance resolution for the VSpec in its sub-tree (e.g., Agent[1…*]); variables, whose resolution requires providing a value (e.g., *name:String VSpec*); and composite VSpec (*CVSpec*), whose resolution requires resolving the VSpecs inside it (e.g., the *Device Type CV*VSpec).

The root of the tree is *MultiAgentSystem*, which implies the resolution of the VClassifier Agent[1…*] and the *variable name:String*. A VSpec tree has mandatory and optional VSpecs. For example, for each of the resolutions of *Agent*, the *CVSpec Device Type, Reasoning Engine CV, Agent platform CV, Connectivity technology CVand the variable type:String* are mandatory. In CVL notation, this is pointed out in the VSpec tree using solid black lines (see the top of Figure 3). The *CVSpecs Services CV* and *SelfManagement CV* are optional (they are linked using dashed black lines at the top of Figure 3). Some cross-tree constraints of this model are also shown in Figure 3: *Sensor mote IMPLIES Reactive*, which means that if the *Device Type* selected for the agent is a *Sensor Mote*, then the *Reasoning Engine*of the *Agent* must be *Reactive*.

**Figure 4:**
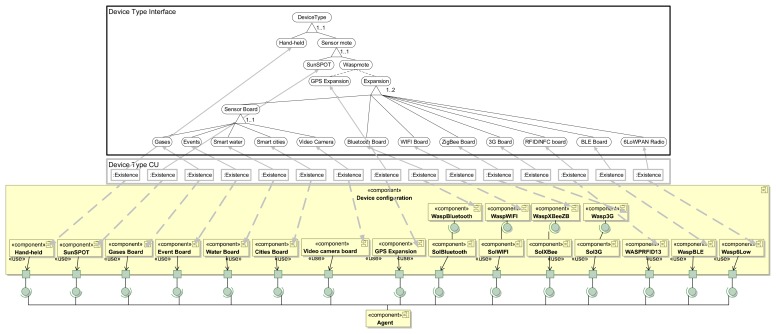
Domain engineering of the device type concern using CVL and Unified Modeling Language (UML).

The next step is to link (or relate) the variability model with the components of the multi-agent system architecture (*i.e.*, the base model). This is done using bindings that link the variation points of the variability model to the UML design of the Multi-agent system architecture. We have represented binding variation points using dashed grey lines; however, this does not mean that linked architectural components or VSpecs are optional. For example, in Figure 3, the device type concern is modeled by *Device Type CV* CVSpec, which is linked to the *Device Type CU* variation point. Finally, this variation point is linked to the *Device Configuration* architectural component (see grey arrows in Figure 3). CVL allows dividing and modularizing the complete variability model at different levels of detail. Thus, it is possible to model each feature of the agent separately in different variability models (like in Figure 4) and, afterwards, to define a complete variability model by relating those models, including all of the features of the agents with their dependency relationships.

### 5.3. IoT Multi-Agent System Architecture

One of the most important benefits of CVL is that the product line architecture can be specified in any MOF-compliant language. In our approach, we have used the UML standard. In the full version of our architecture (see Figure 3), each concern of our multi-agent system for the IoT is modeled with a composite component, and the inter-dependencies are modeled using provided required interfaces. The architecture of our agent follows the mediator pattern [37], so the interaction of the different concerns is encapsulated in the agent component. Our agent requires functionality to provide *Services*, a *Device Configuration* to work and an *Agent Platform* to communicate and locate other agents. Additionally, our agent requires components for *Reasoning* and for accomplishing *Actions*, to monitor the environment and its internal functioning (*Monitoring*), for self-managing (*SelfManagement*) and for weaving aspects in some configurations of the SPL (*Weaving service*). The interaction and dependencies of most of these components are managed by the Agentcomponent with the exception of the dependencies between the *Agent Platform*, which requires the services of the *Connectivity Technology* and *Services* component, which, in turn, require the *Weaving service* and *vice versa*.

The detailed architecture of *Device configuration* is depicted in Figure 4, where each concern (e.g., *Hand-held*) is modeled with a UML component, and its dependencies are modeled using classical, provided required interfaces. Self-StarMAS agents have different options for the configuration of the device in which the agent is embedded. Agents can be embedded in *Hand-held* devices, *SunSPOT* sensor motes and *Waspmote*. *Hand-held* and *SunSPOT* do not have special options for their configuration. However, we model the architecture of Libelium waspmote that considers expansions for different networks technologies (e.g., *WaspWiFi, WaspBluetooth* or *WASPRFID13*) and sensor boards (e.g., *Gases Board or Cities Board*). In addition, *Device configuration* includes components to communicate the agent with the agent platform using available technologies, *SolBluetooth, Sol3G, SolWiFi* and *SolXBee*. These components require services provided by the corresponding component that encapsulates the technology used to communicate with the agent platform. For instance, *SolBluetooth* requires functionality provided by *WaspBluetooth*.

## 6. Application Engineering

This section describes the second phase of our process (located in the center of Figure 2), which generates a valid configuration (customization) of the variability model of the multi-agent system for the IoT. A valid configuration can be seen as an instantiation of an IoT system that satisfies a set of specific requirements. The resultant model is generated taking as input the specific requirements of an application. In order to illustrate this phase of our development process, we use, as a case study, a smart shopping center to enhance the shopping experience.

Our smart shopping center uses different technologies to improve the shopping experience of customers through an active environment. To become an active environment, the physical space is endowed with a set of small devices, namely beacons (iBeacons [38]), that can send signals to smartphones and other personal devices entering their immediate vicinity. Signals, which contain information about the context, are sent via Bluetooth Low Energy Technology (BLE for short). Bluetooth Low Energy [39] is a part of the Bluetooth 4.0 specification released back in 2010. It has a different set of protocols from “classic” Bluetooth, and devices are not backwards-compatible. Accordingly, you can now encounter three types of Bluetooth support: Bluetooth (devices supporting only the “classic mode”); Bluetooth Smart Ready (devices supporting both “classic” and LE modes); and Bluetooth Smart (devices supporting only the LE mode). Beaconing can be applied in all kinds of valuable ways. To put it more simply, beacons transmit data to devices that are in range to enable transmission (immediate, near or far) and allow the user to be located in places where GPS is not as useful, for example, in a museum, park or shopping center. For shopping centers in particular, beacons are important, because they allow a more precise targeting of customers in premises. A customer approaching a store, for example, could receive a message from a battery-powered beacon installed there, offering information or a promotion that relates specifically to products displayed there. In a different area or location of the same store, another beacon transmits a different message. Before beacons, marketers used geofencing technology, so that a message, advertisement or coupon could be sent to consumers when they were within a certain range of a geofenced area, such as within a one-block radius of a store. However, that technology typically relies on GPS tracking, which only works well outside the store. With beaconing, marketers can lead and direct customers to specific areas and products within a particular store or the shopping center itself.

**Figure 5:**
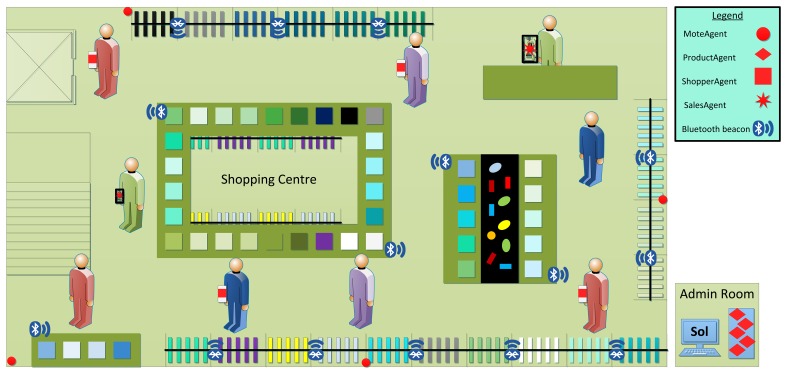
Overview of the multi-agent system of the smart shopping center.

Our case study focuses on a single store (see Figure 5); BLE Beacons are spread over the different elements of the shop associated with specific categories of products and special offers. When a customer with a smartphone is close to a beacon (and also a category of products or a special offer), the device receives a (context-aware) recommendation. The salespeople also benefit from the technology around them. Store employees are informed of the position of customers in the store and receive information of the environmental conditions of the shop in real time on their hand-held devices. This system has been designed as a multi-agent system composed of agents embedded in the different devices that comprise the application. There are agents embedded in sensor motes (which provide the environmental conditions of the store), in the personal devices of the users (both customer and employees) and in the computer of the manager of the shop. In order to enable the communication between these heterogeneous agents, we use the Sol agent platform [23], which is running in the aforementioned computer. These four types of agents in our case study (*MoteAgent, ProductAgent, ShopperAgent* and *SalesAgent*) are used henceforth to illustrate different processes and steps of the application engineering of our SPL process.

### 6.1. Product Configuration

A product configuration is defined in SPL approaches as the set of features that satisfies the application requirements. Therefore, in this process, the software architecture maps the high-level application requirements to the VSpecs included in the VSpec tree. For example, a fundamental requirement of our system is the device where agents are embedded. Therefore, we select those VSpecs featuring personal devices that support Bluetooth connectivity and sensor motes. Smartphones and any other kind of personal device need to support Bluetooth Smart or Bluetooth Smart Ready (and not just Bluetooth) in order to receive the Beacons' tokens. The software architect has to select the VSpec *Device type CV* and choose (also known as resolve) those VSpecs that satisfy the aforementioned requirements. The result is a resolution model for this *CVSpec* (see Figure 4).

In CVL approaches, the product configuration is known as the resolution model (see Section 3), which is created by providing a resolution to the VSpecs (a value in the case of variables, per-instance in the case of the *VClassfiers, etc.*). The configuration is generated by the software architect, assisted by a tool that automatically checks the parent child dependencies and the cross-tree constraints. This automation facilitates the task of the software architect, who does not need to perform this error-prone task manually. Returning to our case study, Libelium waspmotes are used in our system to monitor the environmental conditions of different parts of the shop. Specifically, they are required to measure noise, luminosity, temperature and humidity and send this information periodically to *SalesAgents*. In order to accomplish this task, they are connected to the Sol agent platform using Wi-Fi. The customization options of Waspmotes allow 188 different types of devices. However, in accordance with the cross-tree constraint in Figure 3, (*Noise IMPLIES SmartCities*), *MoteAgent* is deployed in a waspmote with two expansions, *WiFi Board* and *a Sensor Board of the type Smart Cities*. This element is a child of the *group multiplicity Sensor Board*, which, in turn, is a child of the *group multiplicity Expansion*. This type of VSpec (*group multiplicity*) applies some restrictions to the selection of children of a VSpec. In the case of *Sensor Board*, only one of the children can be selected and in the case of *Expansion*, only one or two. These restrictions are due to the physical features of waspmotes, which only allow the plug-in of a sensor board and at most two expansions. Note that the selection of the board *Smart cities* was not initially a requirement of the *MoteAgent*, but, since it is needed to obtain a valid configuration of the VSpec tree, the resolution model must also satisfy its constraints. Therefore, the product configuration not only contains features selected by the architect; in addition, it includes features that result from cross-tree constraints and parent-child dependencies.

### 6.2. IoT Multi-Agent System Architecture Configuration

Once the resolution model has been obtained, the CVL tool generates the resolved model. In our approach, the resolved model is an architecture configuration that contains the set of components and connections that realize features that are part of the resolution model. As stated in Section 3, the variability model in CVL includes the variation points that set out how the variability expressed in the VSpec tree is materialized in the base model. Variation points are bound to elements in the VSpec tree and refer to elements of the multi-agent system for IoT architecture in binding variation points. For instance, the variation point:*Existence* (see Figure 4) bound to *Smart cities* in the *Device Type Interface* VSpec indicates that if *Smart cities* is selected in the resolution model, the *Cities Board* component will exist in the final architecture configuration, and if *Smart cities* is not selected, then this component is not going to be presented in the final architecture.

**Figure 6:**
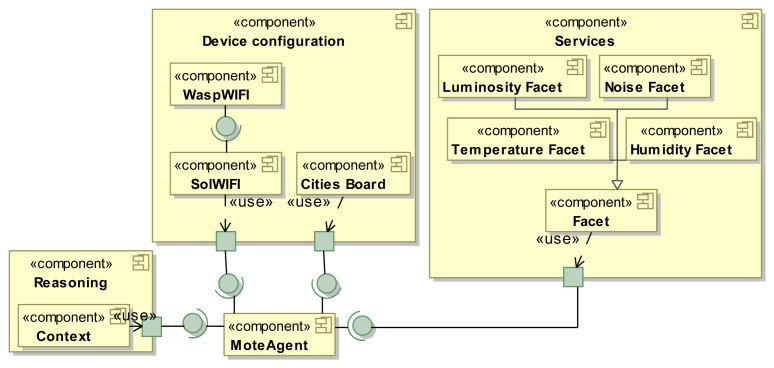
*MoteAgent* architecture configuration for the device configuration concern.

Figure 6 depicts the resolved model for *MoteAgent*. Architectures for sensor motes usually comprise a few components according to the computational and memory capacities of these devices. This architecture configuration includes the architectural components to deal with the monitoring of the luminosity, noise, temperature and humidity. To do so, it has facets to perform tasks for the monitoring (e.g., *Luminosity Facet*) and the *Cities Board* that provide the libraries to perform such tasks. This information is stored in the *Context* component of the *MoteAgent*. Additionally, in order to deploy the agent in the Sol agent platform using the Wi-Fi wireless technology, its architecture includes *WaspWIFI* and *SolWIFI*.

### 6.3. Goal Modeling and Analysis

Goal modeling and analysis are processes that are independent of the product configuration and respond to the need for agent architectures to check consistency between agent goals, context and plans. In this section, we explain how they work and their role in our SPL process.

In agents for the IoT, two important concerns can be identified: the technological infrastructure where the agent is embedded and the application-specific goals. The first is the focus of this paper and can be reused in different application domains (e.g., intelligent transport systems or ambient assisted living). The second varies drastically between applications, and the use of an SPL is not justified. These two concerns in software agents are illustrated by comparing *ShopperAgent* and *SalesAgent* of our case study. Both agents are embedded in hand-held devices (*ShopperAgent* in the smartphones of customers and *SalesAgents* in the hand-held devices of the shop's employees) and deployed in the same agent platform (*i.e.*, Sol). These agents can be developed using our proposal, and their associated resolution models would be very similar. Consequently, several components of the agent architecture could be reused in both agents. However, the set of goals, plans and the knowledge required by the two agents would be different. *ShopperAgent* is intended to provide services to customers outside the shop by enabling clothes to be bought via a mobile application and inside the shops by recommending accessories or clothing that can complement the customer's look with items that are nearby. On the other hand, *SalesAgent* is a tool to help shop attendants monitor the environmental conditions of different areas of the shop and where people get together. Although both agents base their functioning on the use of beacons, *ShopperAgent* is specialized in the recommendation of items and *SalesAgent* in showing the state of the shop.

**Figure 7:**
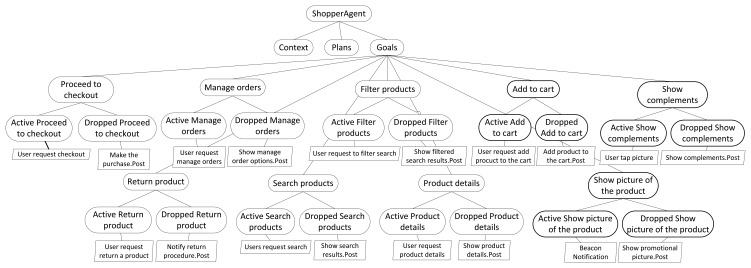
Partial view of the goal model of *ShopperAgent*.

Goal modeling and analysis processes focus on the application specific goals and use the logic-based techniques available in SPL tools, like SPLOT [40], to check consistency and detect conflicts between agent goals. Using SPL tools, we can detect inconsistencies between goals and contexts. Additionally, using resolution models (see Section 3), we can detect conflicts between goals and plans. Therefore, in this case, the SPL is used just as an analysis tool, not for developing the system. This procedure allows the software architect, who is familiar with SPL and CVL, to analyze agent goals without requiring any extra knowledge or external tools. Therefore, this process is only applied to each agent that has a goal-oriented reasoning engine. The output of the goal modeling and analysis processes (the refined goal model in Figure 2) is an SPL that contains a set of goals, plans and context of the agent that is consistent and whose conflicts are detected. Figure 7 depicts a partial view of the goal model of *ShopperAgent*, which follows the semantics of CVL. Due to limitations of space, in Figure 7, only the goals of the *ShopperAgent* are depicted. In the last phase of our development process, the refined goal model is weaved with the IoT multi-agent system architecture configuration to obtain the final application architecture (see Figure 2). To do so, a model-driven development process implemented using the ATLASTransformation Language (ATL) [41] is used [42].

## 7. Related Work

Today SPL technology has been successfully applied to different application areas that include everything from web applications to smart environments [43]. Recently, it has also been applied to multi-agent systems, where the integration of the two technologies is known as MAS-PL (multi-agent system product lines) [8–13].

In [8], Gaia-PL is proposed; it is a modification of the Gaia methodology [44] for the analysis and design of multi-agent systems that uses software product line engineering. Gaia-PL uses a product line perspective to promote reuse in agent-based software systems and enables requirement specifications to be easily reused throughout system development and evolution. To do so, it provides a requirements specification pattern that captures the changing design configurations of agents and the potential reuse of the requirement specifications in future similar systems. The proposal is validated comparing it with the original Gaia methodology, obtaining a 48% reduction in design and documentation times, at least in the case study presented. Unlike our approach, the contributions of this approach only consider the first phase of the SPL process, the domain engineering.

MaCMAS [9] is a methodology that uses formal methods and SPL to model autonomous and self-management properties of multi-agent systems. The work of MaCMAS is based on the use of different models that capture views of the system with different abstraction levels, the relationship between these views and between the agents that compose the system. In this proposal, SPLs are used to model the evolution of the system, taking into account the variations and constraints contained in the SPL. In [9], the authors present a case study in which the modeling of autonomic properties in agents separately is addressed. This issue is also considered in our development process. However, as in [8], this contribution is restricted to the domain engineering of the MAS.

The work presented in [10] focuses on the application engineering process of the SPL, providing an extension of an existing product derivation tool, called GenArch, for the MAS-PL domain. The proposed tool automates the product derivation process of MAS-PL combining the strengths of the code-oriented and the domain-specific configuration knowledge mechanism. The agent architecture model used by this tool is based on previous work on MAS-PL [11] that allows product line engineers to define configuration constraints of agents and agent platforms. The approach is evaluated through the automatic instantiation of two MAS-PLs, demonstrating its potential and benefits to product derivation and configuration knowledge specification. However, although this proposal offers a complete SPL process with tool support, it only considers the generation of Jadex agents [45], which are not suitable for the IoT domain, and it does not consider the development of self-management.

MAS-PL has been exploited in more specific agent domains, like the unmanned aerial vehicles [12]. This approach focuses on the development of safety-critical systems that require certification against specific standards. The authors modify the Product Line on Critical Embedded Systems (ProLiCES) [46] development process for SPL to establish an infrastructure of product line engineering for certified products. The main advantages of the infrastructure proposed are the use of an incremental knowledge base to customize the development process depending on the certification required, fault-tolerance development, as features required for the certification are always included, and reduction of the certification cost. Like the previous proposal, this process focuses on the domain engineering, and although it considers fault-tolerant certification, it does not consider the development of self-management.

All of these approaches are very recent studies and support the use of SPLs to improve the development of multi-agent systems. However, none of the MAS-PL solutions support all phases of the process configuration or address the particularities of the IoT systems, as we do in the present paper. In [9], the authors focus on how to model self-management properties as part of the multi-agent system, as proposed in this paper. However, this contribution does not consider the limitations of IoT devices and is centered on the application level and not on the level of the infrastructure where the agents are embedded.

Let us briefly mention other approaches that benefit from the application of SPL in other domains closely related to IoT, such as robotics and cloud computing environments. With respect to robotics, SPL application is mainly related to the design of industrial robotic systems [13]. SPL has also been applied in this domain to refactor existing mobile robot applications, focusing on source code generation. Closer to our approach, in [47], the authors combine SPL and model-driven development to provide a development and configuration process of robotic systems that explicitly takes into account the variability. Specifically, this approach focuses on addressing the software framework variability and the hardware variability. In [48], previous work is extended to provide support for robotics cloud-computing peculiarities. Furthermore, in this field, different approaches use SPL to customize cloud computing environments at different levels [13].

On the other hand, agents offer a natural metaphor for the development of distributed applications. In addition, agents have similarities with one of the building blocks of the IoT, smart objects [1]. Therefore, they have been previously applied in the development of IoT applications [3–5].

Leppanen *et al.* [5] propose interoperable mobile agents, embedded in smartphones and sensor motes. The idea of the authors is to use a proxy service and representational state transfer principles to move agents between the devices that comprise the IoT system. Using this method, the agent can display the information of sensor motes and hand-held devices to the web using Wi-Fi-based communication. The authors demonstrate the feasibility with a working prototype implemented in Android.

In the approach in [3], an agent-based architecture for the IoT is presented based on the idea of smart object-based IoT applications. The authors propose a layered architecture where agents are embedded in mobile phones, Sun SPOT sensor motes and desktop computers. An intermediate layer enables the communication between the agents and the sensor motes. The authors consider multiple agent technologies, like Jade [49], Jadex, Jade-Leap and Mobile Agent Platform for Sun SPOT (MAPS) [50]. In this case, the agents are the managers of the sensors and make these data available to the IoT system.

The work in [4] presents a generic system architecture implemented in Jade-Leap agents for the IoT that focuses on the domain of smart environments. As in [3], they use a collection of agents that stands for a node of the IoT system. In order to demonstrate their proposal, they present a smart home temperature control application.

None of the these proposals offer an inter-operable and decentralized solution that considers the variability of the IoT domain. Only the work presented in [5] considers the embedding of the agent in sensor motes, and IP-based communication is only considered for the exchange of information between agents in all of them. In addition, they do not explicitly consider the self-management required by the IoT system.

## 8. Lessons Learned

The work presented here is closely related to our work in MDDof agents for ambient intelligence [24]. In this work, we approached the development of agents for ambient intelligence using model-driven development, the Self-StarMAS agents and the Sol agent platform. In our opinion, IoT and ambient intelligence are closely related fields, and indeed, technologies previously applied to develop ambient intelligence or pervasive systems are currently being applied for the development of applications in the IoT [51,52]. Therefore, we think conclusions drawn by us after using both processes could be interesting for other developers of IoT and/or ambient intelligence applications.

In our previous contribution, our multi-agent system was modeled using the Pineapple Platform Independent Metamodel and then transformed to the Self-StarMAS Platform-Specific Metamodel using a model-to-model transformation. The final application was generated using different model-to-text transformation processes, one for each kind of device involved in the system. This procedure lacks an important concern of IoT applications: the technological infrastructure of the application. A platform-independent metamodel specifies software services and interfaces required by independent software technology platforms. Therefore, these metamodels obviate the dependency between services and technology platforms. This approach was affordable for us when we worked with Android and Symbian devices. Agents embedded in mobile phones can provide similar services and support the same network technologies. The problem arose when we started working with Libelium technologies.

Libelium sensor nodes (*i.e.*, waspmotes) are a highly configurable technology whose services and communication means vary greatly according to the configuration of the device. Therefore, we had to consider the dependency between the devices and the services when we modeled the system using Pineapple. This process was error-prone and required the re-running of the model transformation processes many times. A solution to this issue would be to include restrictions written in the Object Constraint Language [53], but they were impossible to write without losing the independence of the platform-independent metamodel. Our conclusion was that in the presence of a high variability that can be reused between different applications, an SPL approach is the best option to include the domain knowledge required to develop the domain.

In general, we think that our proposal facilitates the development for those that wish to use agents as building blocks of the IoT. Currently, there are agent technologies that can be embedded in devices of the IoT, such as Jade-Leap, Agent Factory Micro Edition [54] or the aforementioned MAPS. However, there are no development processes for them that consider the heterogeneity at the infrastructure level. This is especially problematic in the case of sensors due to the great variability present in this domain. Agents for sensors are usually mobile agents that are not intended to interact with agents embedded in other devices. In the case of Agent Factory Micro Edition, it presents a non-mobile agent for Sun SPOT sensor motes, but it is not able to interact with other types of agents of this proposal. In the case of [5], the authors consider a mobile agent that can be translated between sensors and hand-held devices, but only when WIFI communication is available. Therefore, in order to integrate sensor nodes in an agent-based application, developers must develop specific purpose solutions. This is not the case of our proposal, which considers sensor nodes and their heterogeneity from the earliest stages of our development process.

## 9. Conclusions

In this paper, we have presented an SPL process for the development of a multi-agent system in the IoT. The contribution of this approach is a mechanism that promotes the reuse of architectural components to develop IoT systems based on agents. We have implemented our process using the CVL proposed standard. As a part of the process, we have defined a variability model and a multi-agent system architecture that considers the variability present in the IoT domain using a MAS-PL approach.

This initial approach is the first step towards enhancing the development of self-management systems for the IoT, using software agents. Our goal is to perform the reconfiguration of heterogeneous and interconnected devices and the evolution of the system, even at runtime, using agents and the advanced software engineering techniques that variability modeling allows.

Currently, we are assessing the effort required to develop systems using Self-StarMAS agents with our previous method based on MDD [24] and with the SPL process presented in this paper. In our ongoing work, we are considering applying dynamic software product lines to enhance the self-management of the agents at runtime, particularly those embedded in Android devices. Another issue that we have left for future work is the security. This concern is especially important in applications that manipulate personal information or private data. In our proposal, security can be incorporated using two mechanisms: the self-management properties that agents incorporate and the aspect orientation.
